# Activation of short-chain ketones and isopropanol in sulfate-reducing bacteria

**DOI:** 10.1186/s12866-021-02112-6

**Published:** 2021-02-16

**Authors:** Jasmin Frey, Sophie Kaßner, Dieter Spiteller, Mario Mergelsberg, Matthias Boll, David Schleheck, Bernhard Schink

**Affiliations:** 1 Department of Biology, University of Konstanz, 78457 Constance, Germany; 2 Institute of Biology, Albert-Ludwigs-Universität, Freiburg, 79104 Freiburg, Germany

**Keywords:** Anaerobic acetone degradation, Ketone degradation, Pentanone, Sulfate reduction, 2-hydroxyisobutyryl-CoA, Thiamine diphosphate, Adenosylcobalamin

## Abstract

**Background:**

Degradation of acetone by aerobic and nitrate-reducing bacteria can proceed via carboxylation to acetoacetate and subsequent thiolytic cleavage to two acetyl residues. A different strategy was identified in the sulfate-reducing bacterium *Desulfococcus biacutus* that involves formylation of acetone to 2-hydroxyisobutyryl-CoA.

**Results:**

Utilization of short-chain ketones (acetone, butanone, 2-pentanone and 3-pentanone) and isopropanol by the sulfate reducer *Desulfosarcina cetonica* was investigated by differential proteome analyses and enzyme assays. Two-dimensional protein gel electrophoresis indicated that *D. cetonica* during growth with acetone expresses enzymes homologous to those described for *Desulfococcus biacutus*: a thiamine diphosphate (TDP)-requiring enzyme, two subunits of a B_12_-dependent mutase, and a NAD^+^-dependent dehydrogenase. Total proteomics of cell-free extracts confirmed these results and identified several additional ketone-inducible proteins. Acetone is activated, most likely mediated by the TDP-dependent enzyme, to a branched-chain CoA-ester, 2-hydroxyisobutyryl-CoA. This compound is linearized to 3-hydroxybutyryl-CoA by a coenzyme B_12_-dependent mutase followed by oxidation to acetoacetyl-CoA by a dehydrogenase. Proteomic analysis of isopropanol- and butanone-grown cells revealed the expression of a set of enzymes identical to that expressed during growth with acetone. Enzyme assays with cell-free extract of isopropanol- and butanone-grown cells support a B_12_-dependent isomerization. After growth with 2-pentanone or 3-pentanone, similar protein patterns were observed in cell-free extracts as those found after growth with acetone.

**Conclusions:**

According to these results, butanone and isopropanol, as well as the two pentanone isomers, are degraded by the same enzymes that are used also in acetone degradation. Our results indicate that the degradation of several short-chain ketones appears to be initiated by TDP-dependent formylation in sulfate-reducing bacteria.

**Supplementary Information:**

The online version contains supplementary material available at 10.1186/s12866-021-02112-6.

## Background

Short-chain ketones are used in industry as solvents and as precursors for chemical syntheses. Acetone is produced by a wide range of industrial processes, such as the co-production with phenol in the so-called cumene process. It is also produced by fermenting bacteria such as *Clostridium* spp., or in higher animals and humans during ketosis [1, 2]. Isopropanol and butanone are commonly used as solvents in industrial processes and are also side products of microbial degradation processes in soils. Acetone, isopropanol, butanone and 2-pentanone belong to the ten most abundant volatile organic compounds (VOCs) in agricultural soils [3]. Next to acetone and butanone, 3-pentanone, also known as diethyl ketone, is a common odor component of landfills [4]. They are released into the environment in considerable amounts, rendering their microbial degradation a relevant component of the biogeochemical carbon cycle. Acetone (and other ketones) are present in the human body in blood, urine and exhaled air (ketosis), but they are also present in the human intestinal system as a result of microbial fermentation: ketones are the second largest class of volatile organic compounds in human feces [5, 6]. Therefore, degradation of ketones might be relevant also in the metabolism of human gut microbiota. Other sources of acetone and butanone are the emission from plant material during photochemical processes [7, 8].

Several metabolic routes are known for acetone degradation by microorganisms. Aerobic microbes may initiate acetone degradation by activation through a Baeyer-Villiger monooxygenase to methyl acetate ester, by terminal hydroxylation to acetol (hydroxyacetone), or by carboxylation to acetoacetate [6, 9, 10]. Nitrate-reducing and phototrophic bacteria activate acetone by carboxylation which is coupled to the consumption of two or more ATP equivalents per molecule of acetone [11–15]. While such an energy-expensive activation reaction is possible for nitrate-reducing and phototrophic bacteria, it is not feasible for sulfate-reducing bacteria due to their much smaller energy budget [11, 16–18].

Sulfate reducers play important roles in the environment, especially in marine sediments and in technical settings such as the oil industry or wastewater technology [19]. They are also active in the digestive system of animals and humans both in healthy individuals and in persons afflicted with diseases like ulcerative colitis [20, 21]. They also appear to be involved in or associated with chronic inflammatory bowel diseases (IBD) in humans caused most likely by release of highly toxic and reactive hydrogen sulfide, e.g., through H_2_S-mediated inhibition of butyrate oxidation in colonocytes [22–24].

Thus far, two sulfate-reducing bacteria (SRB) have been described that are capable of utilizing acetone and butanone as electron donor and carbon source: *Desulfococcus biacutus* strain KMRActS (DSM 5651) and *Desulfosarcina cetonica* strain 480 (DSM 7267) [16, 25–27]. In *D. biacutus* acetone carboxylase activity was not detectable in cell-free extracts, and the respective candidate genes not found in the genome either. Moreover, no acetoacetate-activating enzyme (e.g. acetyl-CoA:acetoacetate CoA transferase or acetoacetate CoA ligase) was measurable [16, 26, 28]. Further experiments led to the assumption that carbon monoxide may be used as co-substrate leading to a reactive aldehyde species that is subsequently transformed to acetoacetyl-CoA [29]. Genomic and proteomic studies revealed a gene cluster in *D. biacutus* that is strongly induced during acetone utilization: the cluster encodes a thiamine diphosphate (TDP)-dependent enzyme, a B_12_-dependent isomerase and a NAD^+^-dependent dehydrogenase [28]. Results from recent studies suggested that the three (potential) enzymes catalyze the following reaction sequence [30]: (i) formylation of acetone to the branched-chain CoA ester 2-hydroxyisobutyryl-CoA, most likely mediated by the TDP-dependent enzyme, (ii) conversion to 3-hydroxybutyryl-CoA by the B_12_-dependent mutase, and (iii) oxidation to acetoacetyl-CoA by a dehydrogenase. While the enzyme identities and intermediates for the last two reactions were confirmed in cell-free extracts by mass spectrometry and by heterologously produced enzymes [30], the (predicted) TDP-dependent enzyme reaction remained experimentally inaccessible so far. *D. cetonica* harbors genes that are homologous to those found in *D. biacutus*. Therefore, it appears plausible that both bacteria, and probably more sulfate-reducing bacteria, use the same metabolic strategy for acetone degradation [30].

In the present paper, we describe acetone degradation in *D. cetonica* in order to identify similarities and/or differences to that studied previously in *D. biacutus* by differential proteomics, enzyme assays, and CoA ester metabolite analyses. In addition, degradation of butanone (2-methyl ethyl ketone), 2-pentanone, 3-pentanone, and isopropanol (2-propanol) by *D. cetonica* was examined. In this work, also the first description of pentanone degradation by a sulfate reducer is presented on the basis of proteomic data.

## Methods

### Chemicals

Chemicals were purchased from Sigma-Aldrich (Germany), Apollo Scientific (UK), AppliChem (Germany) or Carl Roth GmbH (Germany). The CoA esters were synthesized using the acyl thiophenyl esters as precursors as described earlier [30].

### Bacterial growth conditions

*Desulfosarcina cetonica* strain 480 was cultivated in N_2_/CO_2_ (80%/20%)-flushed, butyl rubber-stoppered bottles containing sulfide-reduced, bicarbonate-buffered medium [25]. The medium was supplemented with 10 mM (or 20 mM in case of pentanones) Na_2_SO_4_ as electron acceptor and 5 mM carbon source (acetone, butyrate, isopropanol, butanone, 2-pentanone, and 3-pentanone). Cultures were incubated at 30 °C in the dark.

### Preparation of cell-free extracts (CFE)

Cells of *D. cetonica* were harvested by centrifugation (8200×*g*, 30 min, 4 °C) and washed twice with Tris–HCl buffer (20 mM, pH 7.2). The cell pellet was resuspended in Tris–HCl buffer (20 mM, pH 7.2) supplemented with 0.5 mg DNase mL^− 1^ and 10 μL mL^− 1^ of Halt™ Protease Inhibitor Cocktail (with EDTA; Thermo Scientific). Cells were disrupted by three to five passages through a cooled French pressure cell at 140 MPa. Cell debris was removed by centrifugation (27,000×*g*, 30 min, 4 °C) to produce cell-free extract (CFE). Membrane fragments were separated by ultracentrifugation (50,000×*g*, 60 min, 4 °C); the supernatant was termed soluble protein fraction. Membrane fragments were washed once with buffer (the remaining supernatant was termed the wash fraction) and resuspended in the same buffer.

### Two-dimensional polyacrylamide gel electrophoresis (2D-PAGE) and proteome analysis

2D-PAGE of soluble proteins was performed using a BioRad Ready Strip IPG/Protean II system. The soluble protein fraction was desalted by Illustra NAP-25 columns (GE Healthcare, Germany) and each sample of 4 mg total protein was precipitated overnight at − 20 °C by addition of 4 volumes of ice-cold acetone. Precipitated protein was collected by centrifugation (10,000×*g*, 10 min, 4 °C) and air-dried at room temperature. The dried protein pellet was solubilized in rehydration buffer (350 μL) and loaded onto an isoelectric focusing (IEF) strip (BioRad IPG strips, 17 cm, pH 4–7) [31]. The isoelectric focusing program involved a voltage ramp (rapid) to a maximal voltage of 10,000 V for at least 3 h and a total focusing of 60,000 Volt-hours (Vh). Strips were equilibrated in SDS equilibration buffers I and II (with DTT and iodoacetamide, respectively) as described earlier [28] and placed onto a 12% SDS-PAGE gel. Gels were stained by colloidal Coomassie staining with (final concentrations) 2% H_3_PO_4_, 10% (NH_4_)_2_SO_4_, 20% methanol, and 0.08% (w/v) Coomassie Brilliant Blue R-250 [32].

Protein spots of interest were excised from the gels and analyzed by peptide fingerprinting-mass spectrometry by the Proteomics Facility of the University of Konstanz [28]. For total proteomic analysis of cell-free extracts (CFE) from cells grown with the different substrates (acetone, butyrate, isopropanol or butanone, respectively), CFEs were analyzed directly with high-resolution peptide fingerprinting-mass spectrometry (LTQ-Orbitrap, Thermo Fisher) by the Proteomics Facility of the University of Konstanz [33].

### Enzyme assays

Activities of key enzymes of acetone degradation were tested by discontinuous assays analyzed by HPLC. All enzyme assays were performed under strictly anoxic conditions (N_2_-flushed 4-ml glass vials sealed with butyl rubber stoppers) in 25 mM MOPS buffer, pH 7.2, (mutase) containing 1 g l^− 1^ NaCl, 0.6 g l^− 1^ MgCl_2_ × 6 H_2_O and 3 mM DTT. B_12_-dependent reaction mixes contained additional 50 μM adenosylcobalamin and were incubated in the dark at 30 °C. Reactions were started by addition of cell-free extract and were followed discontinuously by HPLC-UV or HPLC-MS measurements. Substrate addition and sampling (150–200 μL per sample) under anoxic conditions was performed with gas-tight syringes (Hamilton AG, Switzerland). Samples were mixed thoroughly with dichloromethane to stop the reaction and remove protein. After centrifugation (16,000 x *g*, 5 min, RT (20–23 °C)), the aqueous phase was used for analysis by HPLC or LC-MS.

All photometric assays were carried out in MOPS buffer under strictly anoxic conditions as described above. 3-hydroxybutyryl-CoA dehydrogenase was measured photometrically as 3-hydroxybutyryl-CoA-dependent NAD^+^ reduction at 340 nm. A coupled photometric assay for measurement of the B_12_-dependent isomerization of 2-hydroxyisobutyryl-CoA to 3-hydroxybutyryl-CoA was performed in a similar setup containing 2-hydroxyisobutyryl-CoA instead of 3-hydroxybutyryl-CoA. The subsequent oxidation of the isomerization product 3-hydroxybutyryl-CoA was measured photometrically as described above.

### High pressure liquid chromatography (HPLC) and HPLC- mass spectrometry (MS) measurements

CoA esters were analyzed by HPLC using a Kinetex PFP column (5 mm, 100 A°, 250 3 4.6 mm; Phenomenex, USA) on a Shimadzu Prominence system with PDA detector (SPD-M20A). The temperature was set to 40 °C and a flow rate of 0.75 mL min^− 1^ was used. The injection volume was 5 μL. 100 mM ammonium acetate (eluent B) and acetonitrile (eluent A) were used as eluents. The separation started with 5% eluent A for 15 min followed by a gradient step (1 min) up to 80% eluent A, holding 80% eluent A for 1 min, an additional gradient (1 min) back to 5% eluent A and a re-equilibration with 5% eluent A for 8 min. For HPLC-electrospray ionization (ESI)-MS/MS, an Agilent 1100 HPLC system and the Kinetex PFP column (see above) connected to an LCQ ion trap mass spectrometer (Thermo Fisher Scientific) was used [30]. The injection volume was 50 μL. The column was run isocratically with 95% 100 mM ammonium acetate and 5% acetonitrile for 10 min at a flow rate of 0.75 ml/min and a temperature of 40 °C.

### CoA ester extraction from intact cells and UPLC-MS analysis

For analysis of the CoA ester pool in growing cells, *D. cetonica* was grown with acetone or butyrate as sole carbon source. Cells were harvested in the mid-exponential growth phase, lysed on ice with an ice-cold solution containing 0.1 M formic acid and 80% acetonitrile, and subsequently lyophilized [34]. Lyophilized samples were dissolved in 200 μL 10 mM ammonium acetate and centrifuged two times. The supernatant was used for UPLC-MS measurements.

Samples were analyzed with a Waters Acquity I-Class UPLC coupled to a Waters Synapt G2-Si HDMS ESI-QTOF mass spectrometer. UPLC separation was conducted using a Waters Acquity UPLC HSS T3 column (2.1 × 100 mm) with a 20 min linear gradient of 2% acetonitrile to 40% acetonitrile in 10 mM ammonium acetate buffer at 0.35 ml/min. The mass spectrometer was operated in positive ion mode with a capillary voltage of 3.0 kV, a source temperature of 150 °C, a desolvation temperature of 450 °C and desolvation gas flow of 1000 L/h. Theoretical *m/z* values were calculated and data were evaluated with Waters MassLynx V4.1 SCN916.

## Results and discussion

### General properties of *D. cetonica*

*Desulfosarcina cetonica* is next to *Desulfococcus biacutus* the only described acetone- and butanone-degrading sulfate-reducing bacterium described so far. It was isolated from stratal waters of Apsheron peninsula, Lokbatanskii deposit in Azerbaijan. *D. biacutus* was isolated from anoxic sludge of a sewage plant in Marburg, Germany [16, 27].

In this study, proteome data were analyzed for all major metabolic pathways of *D. cetonica*. A full set of enzymes was identified that allows dissimilatory sulfate reduction and complete oxidation of acetyl residues via the reversed Wood-Ljungdahl pathway. Furthermore, most enzymes of the citric acid cycle (except for malate dehydrogenase and aconitase) were detected by total proteomics.

### Inducible 2-hydroxyisobutyryl-CoA mutase and NAD^+^-dependent 3-hydroxybutyryl-CoA dehydrogenase activity in cell-free extracts of acetone-grown cells of *D. cetonica*

**Fig. 1:**
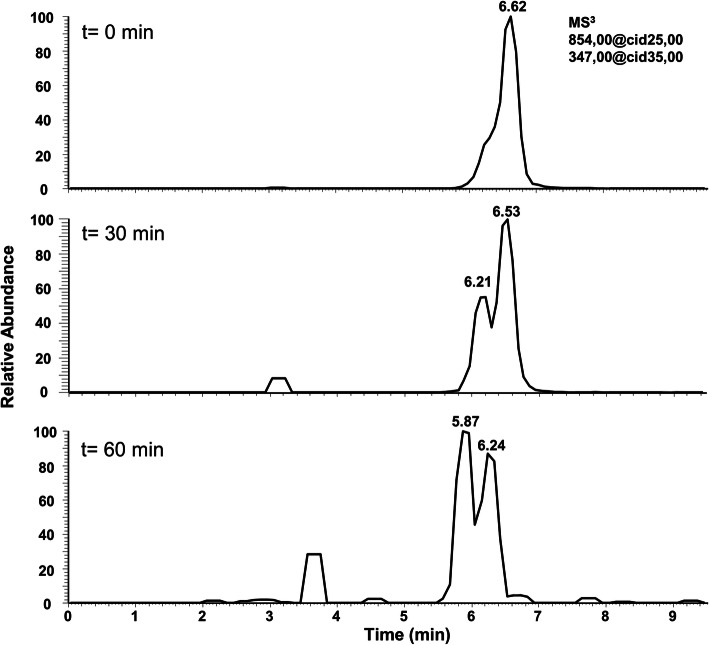
Representative HPLC-MS chromatograms of an in vitro enzyme assay of the B_12_-dependent 2-hydroxyisobutyryl-CoA mutase reaction. Time course measurement of the reaction showing formation of 3-hydroxybutyryl-CoA (peak eluting between 5.87 and 6.21 min), concomitant with disappearance of 2-hydroxyisobutyryl-CoA (peak eluting between 6.24 and 6.62 min). The product 3-hydroxybutyryl-CoA eluted before the substrate 2-hydroxyisobutyryl-CoA

Cell-free extracts (CFE) of acetone-grown cells of *D. cetonica* were analyzed for two key enzyme activities of acetone metabolism observed previously in *D. biacutus*. 2-hydroxyisobutyryl-CoA mutase activity was tested in reactions containing CFE, 2-hydroxyisobutyryl-CoA and B_12_ (adenosylcobalamin) using HPLC fragmentation-mass spectrometry. The B_12_- dependent isomerization of 2-hydroxyisobutyryl-CoA to 3-hydroxybutyryl-CoA was detectable in extracts of cells grown with acetone, but not with butyrate (Fig. 1), suggesting that the synthesis of the enzyme is induced during growth with acetone. Further, the activity of a NAD^+^-dependent 3-hydroxybutyryl-CoA dehydrogenase was detected in CFE of acetone-grown cells (449.6 ± 44.2 mU mg^− 1^); the activity in butyrate-grown cells was lower (222.9 ± 66.4 mU mg^− 1^ protein). When the B_12_-dependent isomerization of 2-hydroxyisobutyryl-CoA was coupled to the NAD^+^-dependent oxidation of 3-hydroxybutyryl-CoA to acetoacetyl-CoA by addition of 2-hydroxyisobutyryl-CoA, B_12_ and NAD^+^, the activity was 1.0 ± 0.2 mU mg^−1^protein in CFE of acetone-grown cells, but no such activity was found in CFE of butyrate-grown cells. This finding agrees with previous studies with *D. biacutus* that was also reported to isomerize 2-hydroxyisobutyryl-CoA to 3-hydroxybutyryl-CoA using a B_12_-dependent mutase followed by dehydrogenation to acetoacetyl-CoA (initially annotated as a 3-oxoacyl-[acyl carrier protein (ACP)] reductase) [28, 30].

**Fig. 2:**
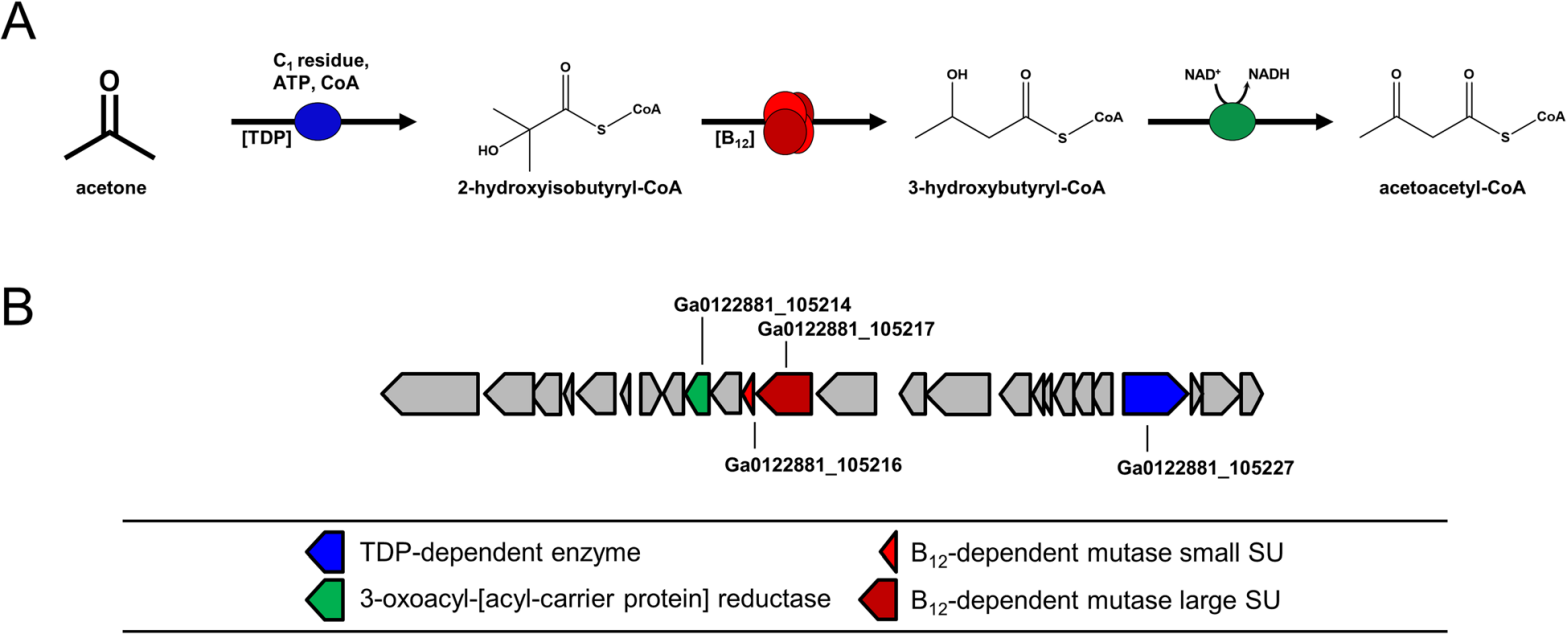
Proposed pathway for acetone degradation in *Desulfosarcina cetonica* strain JCM 12296. **a** Activation of acetone by a yet unknown C_1_-residue, followed by 2-hydroxyisobutyryl-CoA mutase and 3-oxoacyl-[acyl-carrier protein] reductase. **b** Structure of the corresponding gene cluster in *D. cetonica*. Corresponding genes/enzymes (IMG locus tags prefix, Ga0122881_) are color-coded

Attempts to demonstrate the predicted TDP-dependent enzymatic conversion of acetone to 2-hydroxyisobutyryl-CoA (Fig. 2A) were unsuccessful with CFE of *D. cetonica*, as previously reported with CFE of *D. biacutus*: reaction of CFE/acetone with the potential C1-cosubstrates CO, CO_2_, formate, formaldehyde, formyl-CoA or oxalyl-CoA as formyl donor with or without addition of TDP, ATP, FAD, FMN, tetrahydrofolate, NAD^+^ and/or NADH as cofactors (see also Material and methods) did not show any formation of 2-hydroxybutyryl-CoA. Therefore, the nature of the formylating C1 unit remains elusive. It is likely that a C1-intermediate of the Wood-Ljungdahl pathway may be used as co-substrates for acetone activation. In former studies with *D. biacutus,* a co-localization of the TDP-dependent enzyme with membrane proteins was proposed [28] but there is no indication for a functional association with the membrane so far.

### Detection of 2-hydroxyisobutyryl-CoA as a metabolite

Freshly harvested, freeze-dried cells of *D. cetonica* grown with acetone were examined for CoA metabolites by HPLC-MS (see Material and methods). Comparison of acetone- and butyrate-grown cells indicated the presence of 2-hydroxyisobutyryl-CoA in acetone-grown cells at about ten-fold higher abundance than in butyrate-grown cells (see Supporting information Fig. S1), thus supporting the proposed pathway (Fig. 2A). However, formyl-CoA as a potential formyl donor for the predicted TDP-dependent conversion of acetone to 2-hydroxyisobutyryl-CoA was not detectable by HPLC-MS in the cells under these conditions. No further prominent CoA-intermediate was found exclusively in acetone-grown cells during analyses of ion chromatograms with CoA ester-specific fragmentation patterns.

### Proteomic identification of acetone-inducible enzymes in *D. cetonica*

**Fig. 3:**
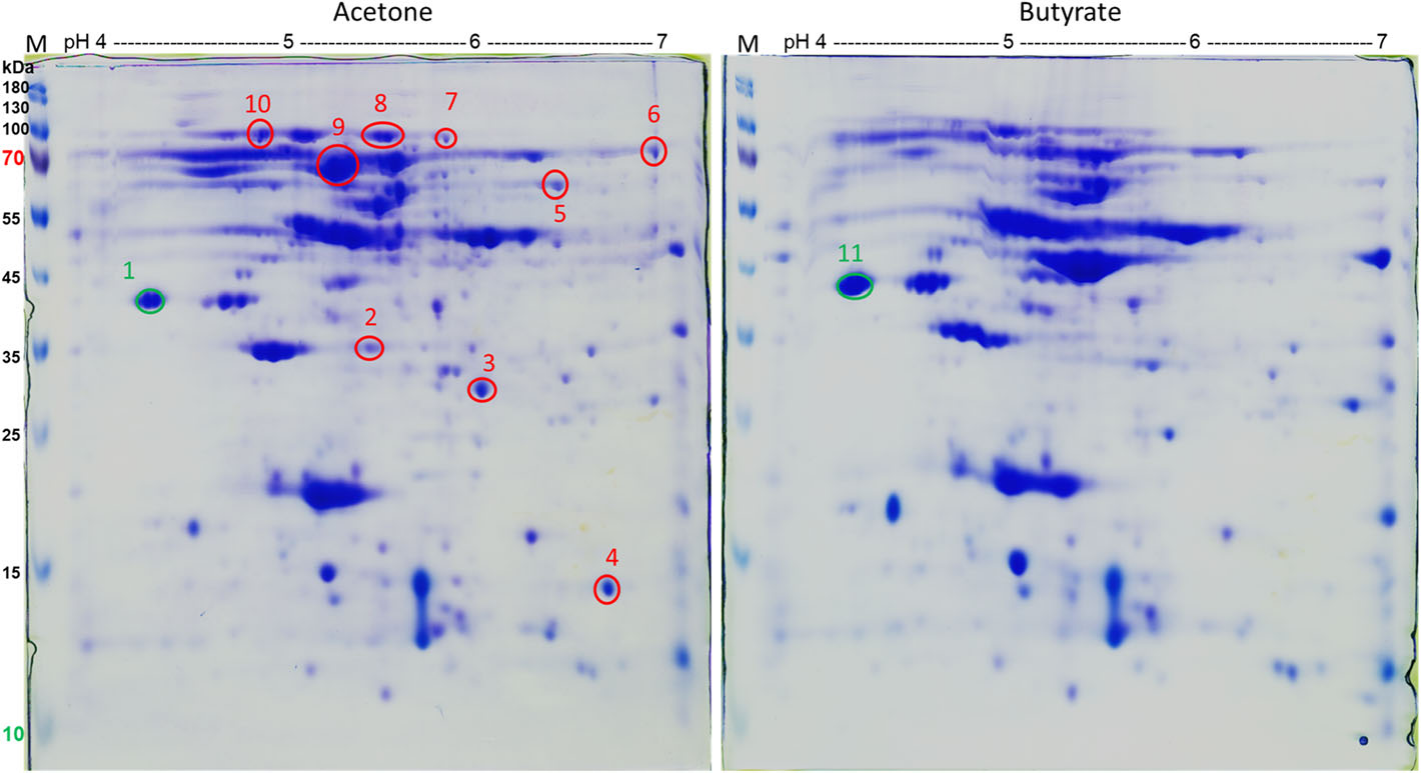
2D PAGE analysis of soluble proteins obtained from acetone- and butyrate-grown cells of *D. cetonica*. Red-labeled spots are acetone-induced, green-labeled spots were of similar size in both samples. Spots were excised and analyzed by peptide mass fingerprinting (for results see Table 1)

Soluble protein fraction of acetone- and butyrate-grown cells were compared by two-dimensional protein electrophoresis (2D PAGE). All protein spots with higher abundance in acetone-grown cells (Fig. 3) were excised, and the tryptic peptides obtained were analyzed by MS. The proteins identified are listed in Table 1. Nine protein spots (spots 2–10, Fig. 3) were either exclusively present or were more abundant in the soluble protein fraction of acetone-grown cells. Two further protein spots produced at almost equal abundance under both growth conditions were excised as controls to ensure correct localization in the gel and exclusion of carry-over of other proteins (spots 1 and 11, Fig. 3).

**Table 1:** Identification of proteins after 2D-PAGE analysis of *D. cetonica* grown with acetone/sulfate vs. butyrate/sulfate. Spots 2–10 were specifically induced after growth with acetone. Proteins of the proposed acetone degradation cluster are marked in **bold**. Spots 1 and 11 served as control spot and were found to be identical in both conditions

Spot ID	IMG locus tag	Annotation	Score	MW	calc. pI
1	Ga0122881_14251	electron transfer flavoprotein beta subunit	2029	26.6	4.35
2	Ga0122881_108331	hypothetical protein	3373	19.3	5.73
3	Ga0122881_105,214	**3-oxoacyl-[acyl-carrier protein] reductase**	23,994	28.0	5.72
4	Ga0122881_105,216	**methylmalonyl-CoA mutase, C-terminal domain**	15,935	14.5	6.54
5	Ga0122881_103346	Formate-tetrahydrofolate ligase	10,064	53.4	6.55
6	Ga0122881_105,227	**Acetolactate synthase large subunit**	1857	77.6	6.62
7	Ga0122881_10451	pyruvate carboxylase subunit B	11,267	63.7	5.73
8	Ga0122881_10843	acetyl-CoA decarbonylase/synthase beta subunit	20,590	81.0	5.35
9	Ga0122881_105,217	**methylmalonyl-CoA mutase**	14,980	64.7	5.11
10	Ga0122881_103051	iron complex outermembrane receptor protein	9818	73.2	4.96
	Ga0122881_11832	outer membrane receptor for ferrienterochelin and colicins	7161	74.0	5.00
11	Ga0122881_14251	electron transfer flavoprotein beta subunit	13,308	26.6	4.35

Spots 3, 4, 6 and 9 were clearly higher abundant in cells grown with acetone (Fig. 3) and are annotated as 3-oxoacyl-[acyl-carrier protein] reductase (IMG locus tag: Ga0122881_105,214; in the following the tag prefix Ga0122881_ is omitted), methylmalonyl-CoA mutase (C-terminal domain) (105216), TDP-dependent acetolactate synthase large subunit (105227), and methylmalonyl-CoA mutase (105217) (Table 1), respectively. The genes for these proteins are located in the same gene cluster (Fig. 2B) and most likely the two proteins annotated as methylmalonyl-CoA mutase (105,216, 105,217) are two different assingments of one enzyme, as described for the homologous proteins in *D. biacutus* [30]. In *D. biacutus,* homologous proteins were observed to be acetone-induced, and the encoding genes are also located in one gene cluster [28]. The acetolactate synthase (105227) has high similarities (87.7% identity at the amino acid level using IMG Genome BLASTP) to an acetone-induced TDP-dependent enzyme of *D. biacutus*. Also the two subunits of B_12_-dependent methylmalonyl-CoA mutase (105,216, 105,217) showed high similarities (85.6 and 85.5%, respectively amino acid sequence identity) to the acetone-induced homologs of *D. biacutus*. The 3-oxoacyl-[acyl-carrier protein] reductase of *D. cetonica* exhibited 82.2% sequence identity at the amino acid level to an acetone-induced enzyme in *D. biacutus.* Furthermore, it was demonstrated that the acetone-specific enzyme initially annotated as 3-oxoacyl-[acyl-carrier protein] reductase of *D. biacutus* oxidizes 3-hydroxybutyryl-CoA to acetoacetyl-CoA [30].

Spots 2, 5, 7, 8, and 10 were identified as a hypothetical protein, formate-tetrahydrofolate ligase, pyruvate carboxylase subunit B, acetyl-CoA decarbonylase/synthase beta subunit and iron complex outer membrane receptor protein / outer membrane receptor for ferrienterochelin and colicins. However, these five proteins appeared to be constitutively expressed under both growth conditions.

In summary, these findings, as well as the identification of 2-hydroxyisobutyryl-CoA in extracts of acetone-grown cells in the metabolomics assay strongly suggest that acetone is degraded in *D. cetonica* through the same pathway as proposed for *D. biacutus* [30].

### Proteomic identification of inducible proteins in *D. cetonica* during growth with isopropanol and butanone

**Fig. 4:**
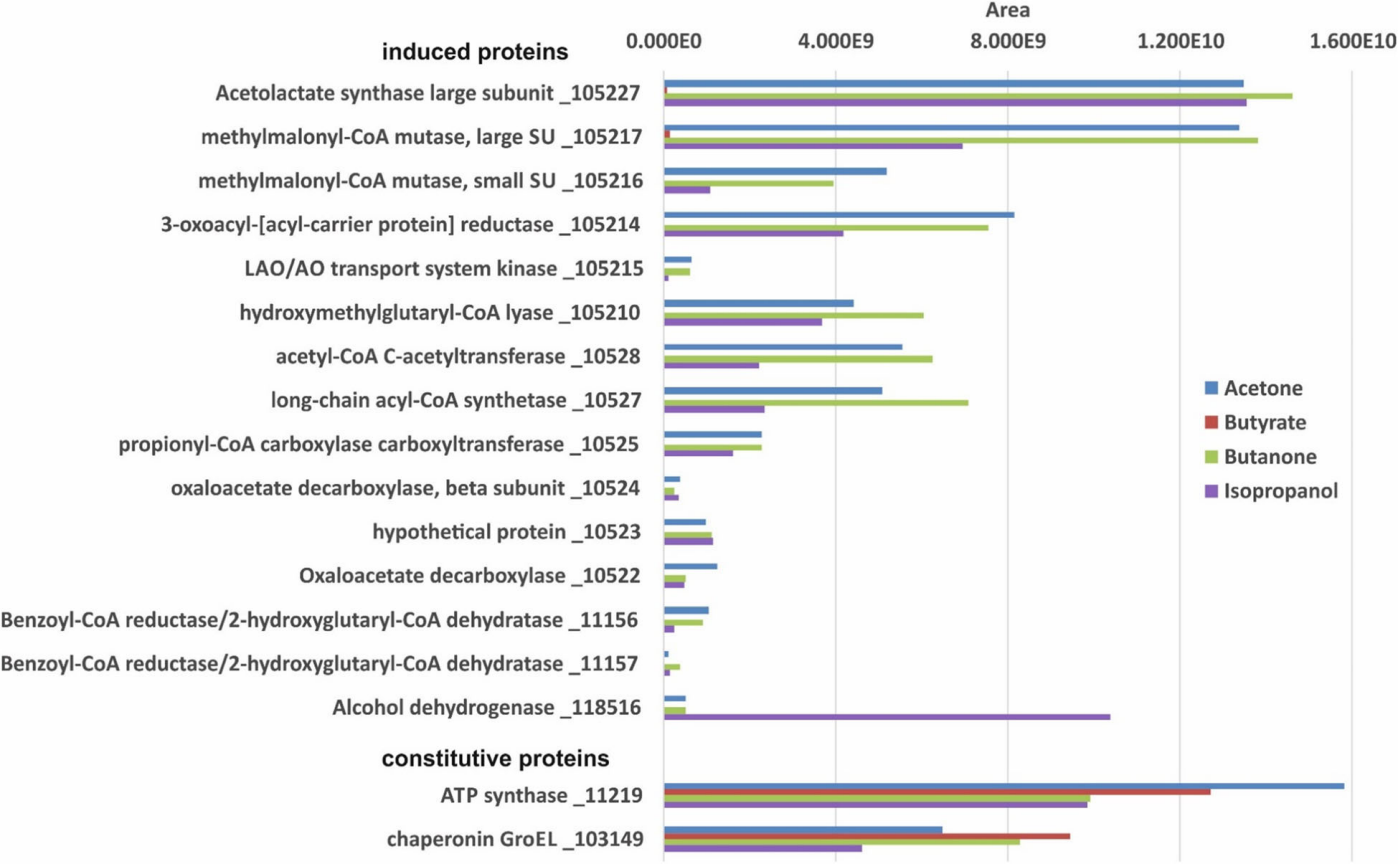
Total proteome analysis of cell-free extracts of *D. cetonica* after growth with different substrates (acetone [blue], butyrate [red], isopropanol [purple] and butanone [green]). Induced and constitutively expressed proteins are shown. IMG locus tag numbers (Ga0122881_number) are given for each protein. Higher area values represent higher protein abundance

We examined the expression patterns of proteins in CFE of *D. cetonica* after growth with four different electron donors, acetone, butanone, isopropanol, and butyrate (control), by total proteomic analysis without preceding 2D-PAGE separation. For these analyses, cultures had been transferred at least 10 times with the respective substrate prior to analysis to ensure adaptation. The data obtained confirmed the proteins that were identified by 2D-PAGE. Several proteins with higher abundance in cells grown with acetone vs butyrate were identified (Fig. 4). A TDP-dependent protein annotated as acetolactate synthase (105227) was highly induced. In addition, the small and the large subunit (105,216, 105,217) of a B_12_-dependent isomerase acting on CoA esters (annotated as methylmalonyl-CoA mutase) and a 3-oxoacyl-[acyl-carrier protein] reductase (105214) were more abundant in acetone-grown cells.

In addition, several proteins abundant in acetone-grown cells were identified that escaped detection during 2D-PAGE and were annotated as: hydroxymethylglutaryl-CoA lyase (105210), acetyl-CoA C-acetyltransferase (10528), long-chain acyl-CoA synthetase (10527), propionyl-CoA carboxylase carboxyltransferase subunit (10525), oxaloacetate decarboxylase beta subunit (10524), a hypothetical protein (10523), oxaloacetate decarboxylase gamma chain (10522), and two subunits of a benzoyl-CoA reductase/2-hydroxyglutaryl-CoA dehydratase (11,156, 11,157). Many of these proteins were found to be acetone-inducible also in *D. biacutus* [28].

All these acetone-specific (compared to butyrate) proteins were found also in CFE of butanone- and of isopropanol-grown cells. With the exception of the two benzoyl-CoA reductase/2-hydroxyglutaryl-CoA dehydratase subunits, all other genes of the respective acetone-induced proteins are located in one gene cluster (see Fig. S2 in supporting information). Additionally, a LAO/AO transport system kinase (105215) related to ArgK (MeaB) was found which may serve as a stabilizing G protein for the B_12_-dependent enzyme [35]. Interestingly, the genes of these six induced proteins (10522–10,528) are clustered together in one gene cluster in which also genes coding for the TDP-dependent enzyme, the two subunits of the B_12_-dependent mutase and the 3-oxoacyl-(ACP) reductase described above are located. This strongly implies that these enzymes are involved in the degradation of acetone, isopropanol, and butanone. One might speculate that these enzymes are needed for production of another precursor molecule (e.g. formyl-CoA) which is used for acetone activation. Moreover, an alcohol dehydrogenase (118516) was nearly 20-fold higher abundant during growth with isopropanol than in acetone- or butanone-grown cells, and was not detected in extracts of butyrate-grown cells. Thus, this enzyme most likely catalyzes the dehydrogenation of isopropanol to acetone, but is encoded in a gene cluster different from that of the genes involved in acetone degradation.

The main difference between *D. biacutus* and *D. cetonica* is that the latter does not contain a homologue of the acetone-inducible threonine dehydrogenase (DebiaDRAFT_04514), and no homologous gene of the respective protein was found in the genome. In an earlier study, this dehydrogenase was proposed to have a detoxifying function, as it utilizes a broad variety of short-chain alcohols, aldehydes, and ketones [36]. One may speculate that lack of this protein could explain the measured difference in growth yields (for *D. cetonica* only around 1/3 compared to *D. biacutus*) between *D. biacutus* and *D. cetonica* growing with the same substrate [16, 25, 26].

The finding that the same enzyme proteins are highly abundant during growth with acetone, butanone, and isopropanol strongly suggests that degradation of these three substrates involves common proteins.

### Differential in vitro activities in cell-free extracts of isopropanol- and butanone-grown *D. cetonica*

Enzyme assays with CFE of isopropanol-grown cells exhibited a specific activity of 35.8 ± 5.1 mU mg^− 1^ protein for reduction of 5 mM acetone with 0.5 mM NADH, and 14.0 ± 1.0 mU mg^− 1^ protein for oxidation of 5 mM isopropanol with 5 mM NAD^+^, whereas no activity (detection limit: < 0.5 mU mg^− 1^ protein) was detected in extracts of butyrate-grown cells. These activities were around 10-fold higher than in acetone- or butanone- grown cells. Activities of NADH-dependent acetone reduction were lower in CFE of acetone- or butanone-grown cells: 3.1 ± 0.3 and 4.9 ± 2.4 mU mg^− 1^ protein, respectively. Enzyme activities of NAD^+^-dependent isopropanol oxidation were below the detection limit in CFE of acetone- or butanone-grown cells.

NAD^+^-dependent oxidation of 3-hydroxybutyryl-CoA was detected in isopropanol- and butanone-grown CFEs with specific activities of 250.1 ± 37.1 and 391.0 ± 76.3 mU mg^− 1^ protein, respectively. *D. cetonica* grows with isopropanol slower and to lower cell densities than with acetone or butanone [27].

*D. biacutus* has been described as well to grow with butanone and isopropanol [16]. Under both growth conditions, also a B_12_-dependent isomerization of 2-hydroxyisobutyryl-CoA to 3-hydroxybutyryl-CoA was detected using a coupled assay with NAD^+^. Here, CFE of isopropanol-grown cells of *D. cetonica* exhibited an activity of 1.8 ± 0.5 mU mg^− 1^ protein and 1.3 ± 0.4 mU mg^− 1^ protein for CFE of butanone-grown cells.

Degradation of butanone via initial formylation would lead to a C5-CoA ester, which may be cleaved thiolytically to acetyl-CoA and propionyl-CoA. Propionate is not excreted to the growth medium during growth with butanone, obviously because *D. cetonica* can grow also with propionate as sole carbon source [27]. Degradation pathways in the sulfate-reducing bacteria *D. biacutus* and *D. cetonica* for acetone, isopropanol and butanone are depicted in Fig. 6A. This strategy of activation may be used also for longer ketones and might reflect a common concept for activation and utilization of these ketones.

### Proteomic identification of inducible proteins in *D. cetonica* during growth with 2-pentanone and 3-pentanone

**Fig. 5:**
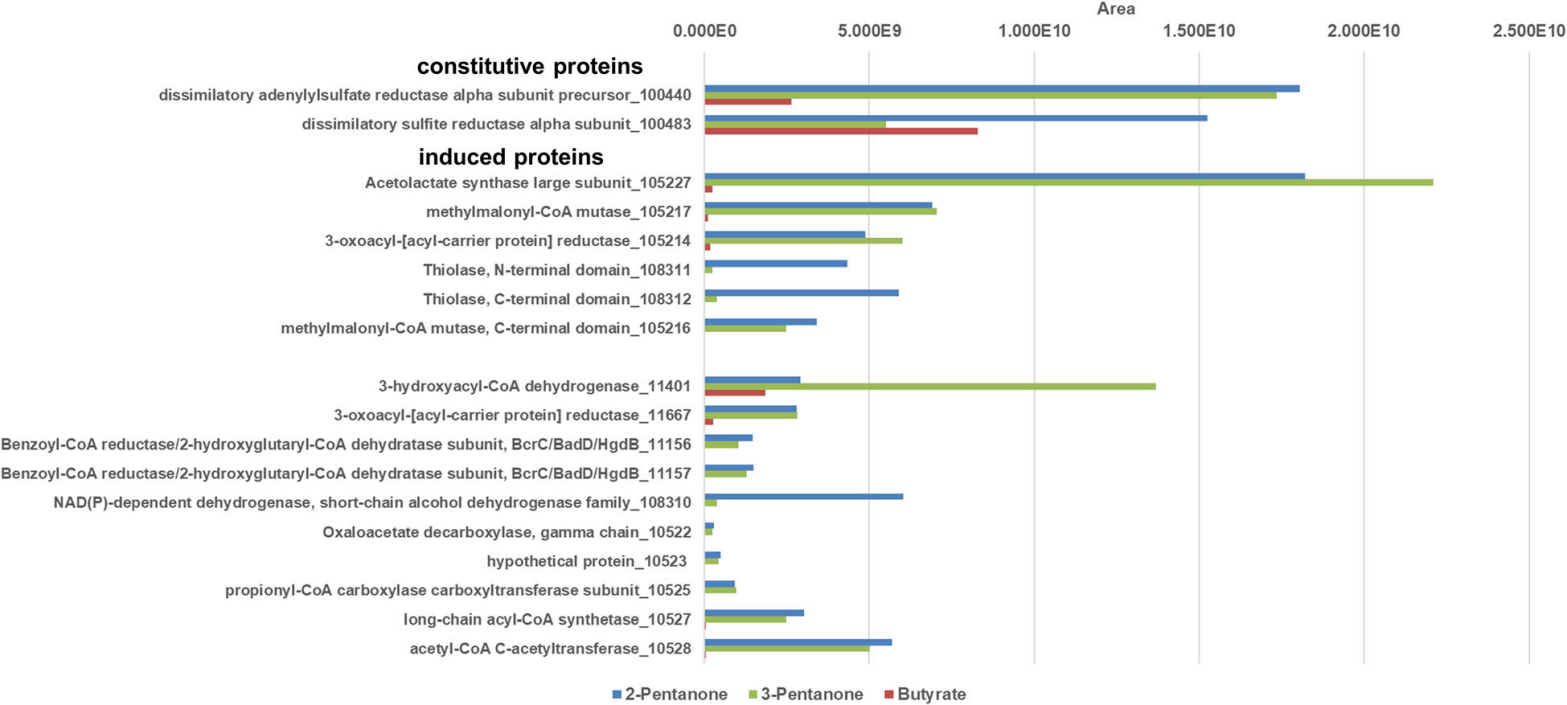
Total proteome analysis of cell-free extracts of *D. cetonica* after growth with different substrates (2-pentanone [blue], 3-pentanone [red] and butyrate [green]). IMG locus tag numbers (Ga0122881_number) are given for each protein. Higher area values represent higher protein abundance

Protein expression patterns after growth with C5-compounds like 2-pentanone and 3-pentanone were studied in comparison to butyrate as control substrate. Proteomic analysis was performed with CFEs of 2-pentanone- and 3-pentanone-grown cells which had been transferred at least 10 times on the respective substrate. Several induced proteins were identified that exhibited enhanced abundance compared to growth with butyrate (Fig. 5).

One of these most abundant proteins is a TDP-dependent acetolactate synthase (105227). Also specifically induced during growth with both pentanones were the small and large subunit (105,216, 105,217) of a B_12_-dependent mutase (annotated as methylmalonyl-CoA mutase) and a 3-oxoacyl-[acyl-carrier protein] reductase (105214).

Further proteins with abundance in 2−/3-pentanone-grown cells were identified such as two subunits of a benzoyl-CoA reductase/2-hydroxyglutaryl-CoA dehydratase (11,156, 11,157), as well as four proteins whose genes are directly adjacent to each other: a propionyl-CoA carboxylase carboxyltransferase subunit (10525), an oxaloacetate decarboxylase beta subunit (10524), a hypothetical protein (10523) and an oxaloacetate decarboxylase gamma chain (10522). Also an acetyl-CoA C-acetyltransferase (10528) and a long-chain acyl-CoA synthetase (10527) were identified to be induced during growth with the two pentanones. All of the above-mentioned proteins were discovered to be highly abundant also after growth with acetone, butanone and isopropanol.

**Fig. 6:**
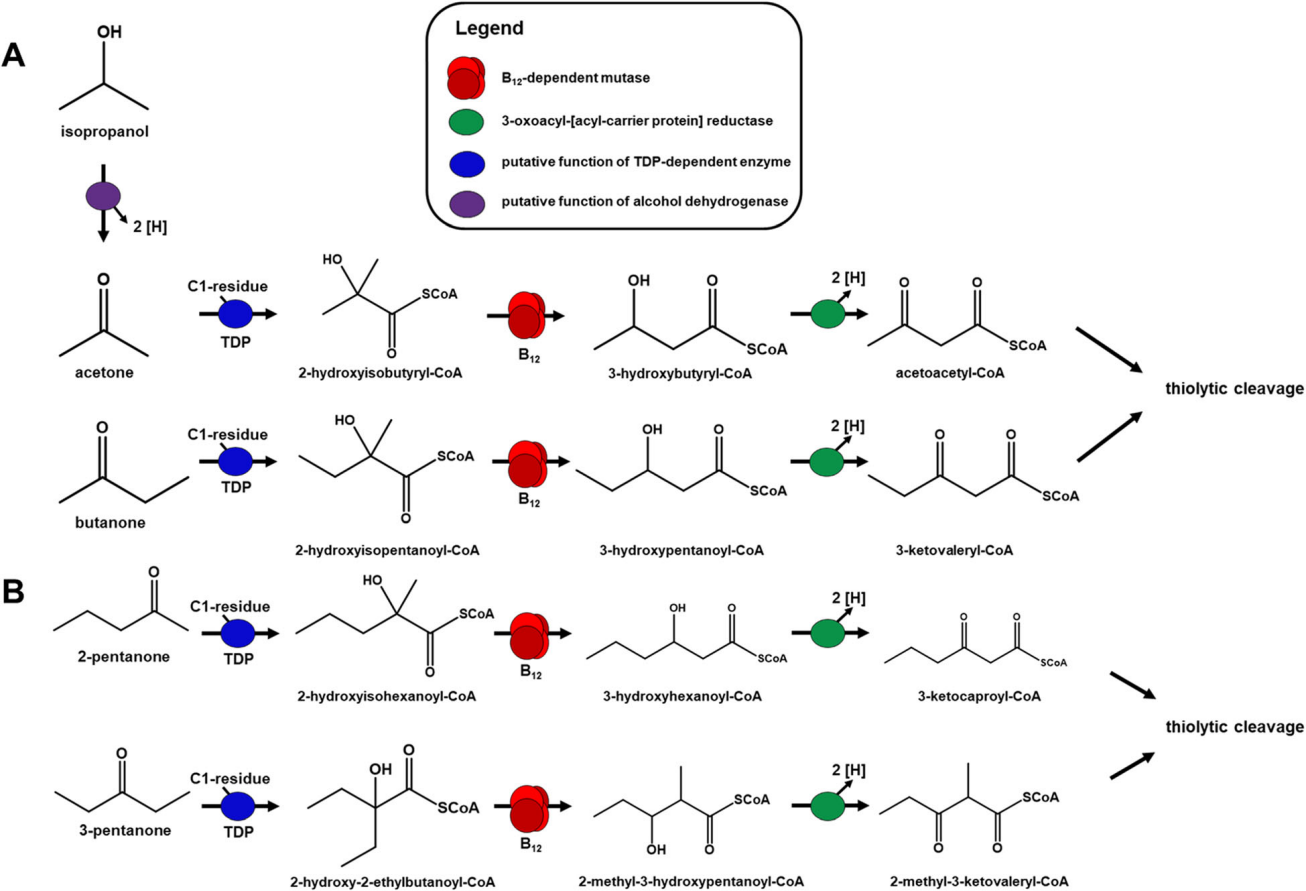
Proposed degradation pathways for acetone, isopropanol and butanone in the sulfate-reducing bacteria *D. biacutus* and *D. cetonica* (Fig. 6**a**) and for 2-pantanone and 3-pentanone in *D. cetonica* (Fig. 6**b**)

The results, although still preliminary, indicate that the two pentanones are degraded analogous to acetone and butanone via different intermediates: Pentanone-2 degradation after formylation and linearization would lead to an acetyl and a butyryl residue, whereas degradation of 3-pentanone would form two propionyl-CoA as intermediates (Fig. 6B). It appears that 2-pentanone is easier to degrade than 3-pentanone, as 3-pentanone cultures need more than 3 times longer to reach the plateau phase (data not shown). One might speculate that the respective enzymes involved may be sterically hindered by the two ethyl residues of 3-pentanone.

## Supplementary Information

**Additional file 1: Figure S1.** HPLC-MS chromatograms from lysates of acetone (Sample Acetone 1–3)- and butyrate (Sample Butyrate 1–3)-grown cells compared to pure standards of 2-hydroxyisobutyryl-CoA (2-HiB-CoA) and 3-hydroxybutyryl-CoA (3-HB-CoA). Acetone-grown cells show substantially more 2-hydroxyisobutyryl-CoA (elutes between 10.30–10.45 min) whereas in butyrate-grown cells mainly 3-hydroxybutyryl-CoA (elutes between 10.48–10.58 min) was identified. **Figure S2.** Gene cluster showing specifically acetone-induced genes in Desulfosarcina cetonica. Most prominent genes are symbolized as red arrows and are annotated as acetolactate synthase (105227), a small and a large subunit of a methylmalonyl-CoA mutase (105,216, 105,217) and a 3-oxoacyl-[acyl-carrier protein] reductase (105214). Green genes are found to be expressed acetone-specifically (also in butanone- and isopropanol-grown, not in butyrate-grown CFE) by proteome analysis. These genes are annotated as an oxaloacetate decarboxylase, gamma chain (10522), a hypothetical protein (10523), an oxaloacetate decarboxylase beta subunit (10524), a propionyl-CoA carboxylase carboxyltransferase subunit (10525), a long-chain acyl-CoA synthetase (10527), acetyl-CoA C-acetyltransferase (10528), as well as a hydroxymethylglutaryl-CoA lyase (105210), a hypothetical protein (105211), a glyoxylase, beta-lactamase superfamily II (105213), LAO/AO transport system kinase (105215), an acyl-CoA hydrolase (105218), an anaerobic selenocysteine-containing dehydrogenase (105220), a two component transcriptional regulator, LuxR family (105224) and a histidine kinase-, DNA gyrase B-, and HSP90-like ATPase (105225).

## Data Availability

All supporting data are presented in the main paper and the supplementary files. The genome annotation of *Desulfococcus biacutus* strain KMRActS and the nucleotide and amino-acid sequences of locus tag DebiaDRAFT_04514 are publicly available within the Joint Genome Institute (JGI) Integrated Microbial Genomes (IMG) system under IMG genome ID 2512047085; the genome sequencing and annotation has been described in ref. [20].
