# Supplementary material for: Activation of short-chain ketones and isopropanol in sulfate-reducing bacteria
Source: BMC Microbiol. 2021 Feb 16;21:50. doi: 10.1186/s12866-021-02112-6 (PMC7888143; doi:10.1186/s12866-021-02112-6)
Supplement: Supplementary file 1 — Additional file 1: Figure S1. HPLC-MS chromatograms from lysates of acetone (Sample Acetone 1–3)- and butyrate (Sample Butyrate 1–3)-grown cells compared to pure standards of 2-hydroxyisobutyryl-CoA (2-HiB-CoA) and 3-hydroxybutyryl-CoA (3-HB-CoA). Acetone-grown cells show substantially more 2-hydroxyisobutyryl-CoA (elutes between 10.30–10.45 min) whereas in butyrate-grown cells mainly 3-hydroxybutyryl-CoA (elutes between 10.48–10.58 min) was identified. Figure S2. Gene cluster showing specifically acetone-induced genes in Desulfosarcina cetonica. Most prominent genes are symbolized as red arrows and are annotated as acetolactate synthase (105227), a small and a large subunit of a methylmalonyl-CoA mutase (105,216, 105,217) and a 3-oxoacyl-[acyl-carrier protein] reductase (105214). Green genes are found to be expressed acetone-specifically (also in butanone- and isopropanol-grown, not in butyrate-grown CFE) by proteome analysis. These genes are annotated as an oxaloacetate decarboxylase, gamma chain (10522), a hypothetical protein (10523), an oxaloacetate decarboxylase beta subunit (10524), a propionyl-CoA carboxylase carboxyltransferase subunit (10525), a long-chain acyl-CoA synthetase (10527), acetyl-CoA C-acetyltransferase (10528), as well as a hydroxymethylglutaryl-CoA lyase (105210), a hypothetical protein (105211), a glyoxylase, beta-lactamase superfamily II (105213), LAO/AO transport system kinase (105215), an acyl-CoA hydrolase (105218), an anaerobic selenocysteine-containing dehydrogenase (105220), a two component transcriptional regulator, LuxR family (105224) and a histidine kinase-, DNA gyrase B-, and HSP90-like ATPase (105225). [file 12866_2021_2112_MOESM1_ESM.pdf]

Activation of short-chain ketones and isopropanol in sulfate-reducing bacteria

Jasmin Frey[1], Sophie Kaßner[1], Dieter Spiteller[1], Mario Mergelsberg[2], Matthias Boll[2], David Schleheck[1] and Bernhard Schink[1*]

[1] Department of Biology, University of Konstanz, 78457 Konstanz, Germany

[2] Institute of Biology, Albert-Ludwigs-Universität, Freiburg, 79104 Freiburg, Germany

* Corresponding author:

E-mail: bernhard.schink@uni-konstanz.de

Tel. +49-07531-88 2140

Fax +49-07531-88 4047

Target journal: BMC Microbiology (Research Article)

Key words: anaerobic acetone degradation, sulfate reduction, 2-hydroxyisobutyryl-CoA, thiamine diphosphate, adenosylcobalamin

Supplementary Information

Acetone-grown cells

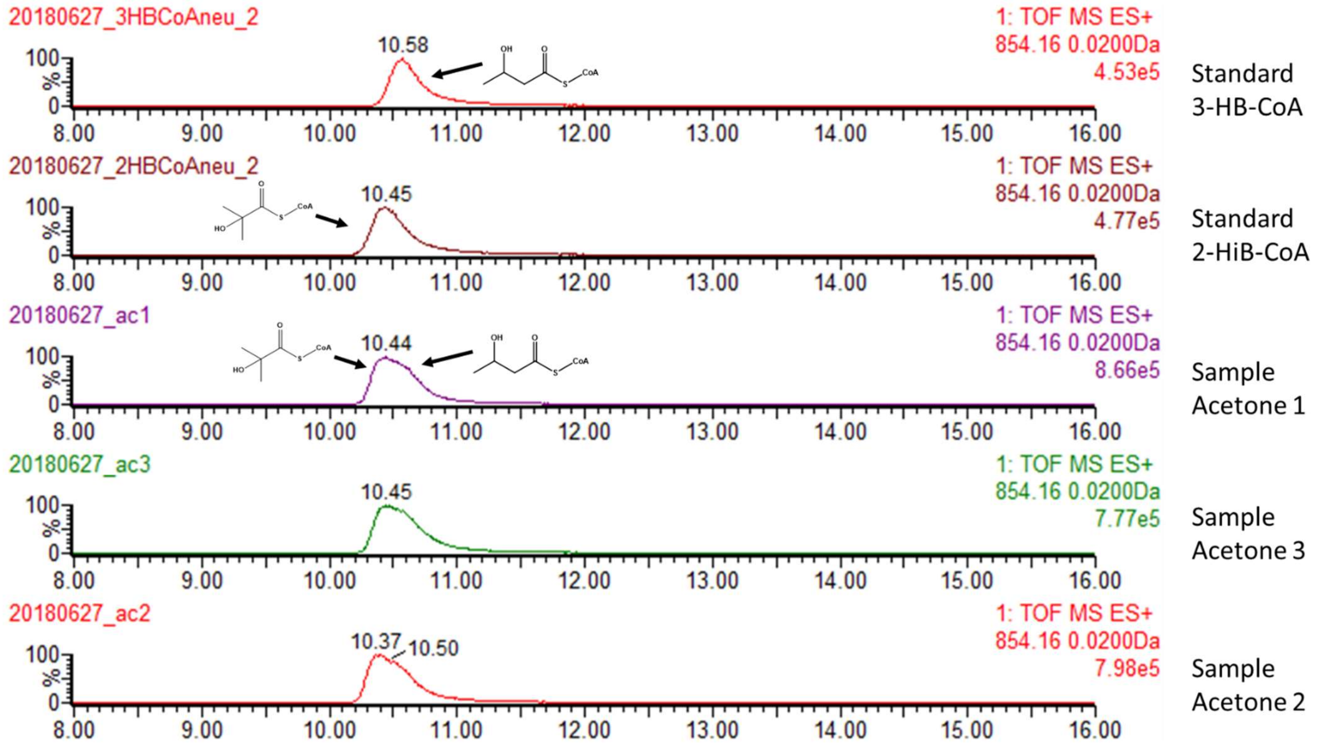

Butyrate-grown cells

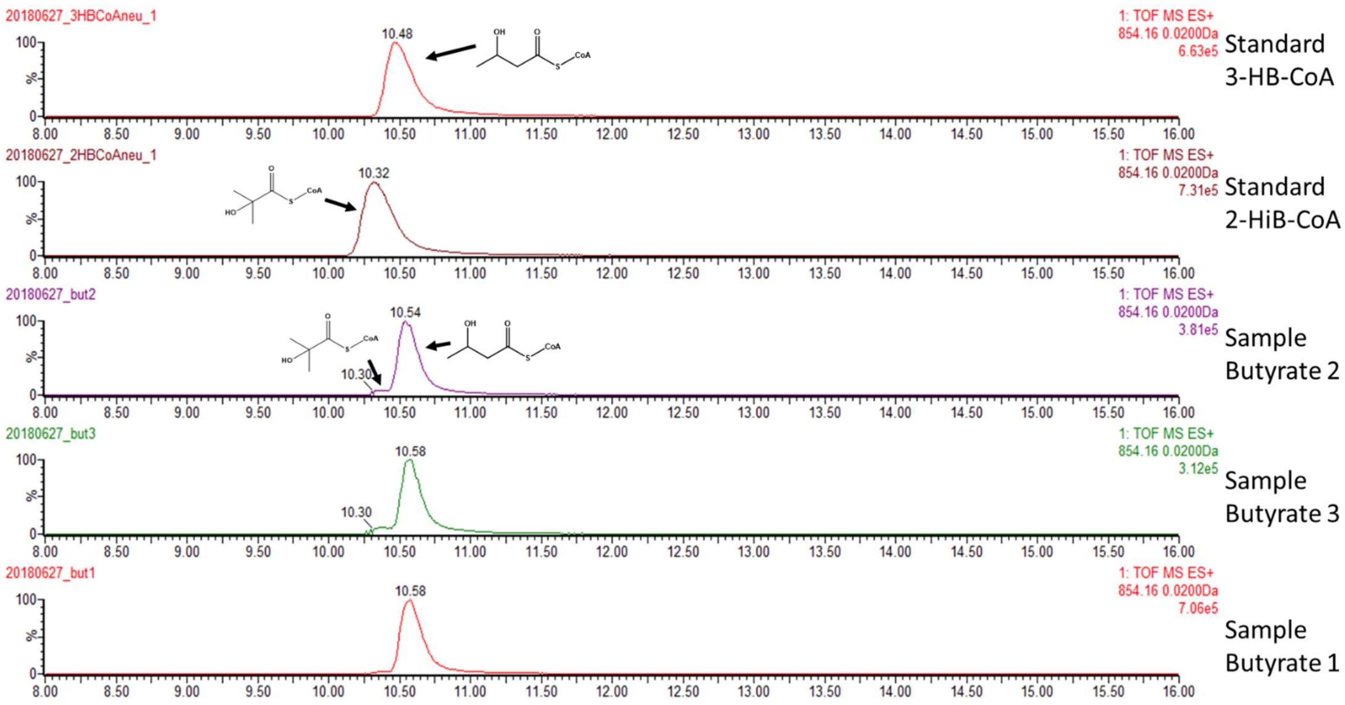

Fig. S1 HPLC-MS chromatograms from lysates of acetone (Sample Acetone 1-3)- and butyrate (Sample Butyrate 1-3)-grown cells compared to pure standards of 2-hydroxyisobutyryl-CoA (2-HiB-CoA) and 3-hydroxybutyryl-CoA (3-HB-CoA). Acetone-grown cells show substantially more 2-hydroxyisobutyryl-CoA (elutes between 10.30 - 10.45 min) whereas in butyrate-grown cells mainly 3-hydroxybutyryl-CoA (elutes between 10.48 - 10.58 min) was identified.

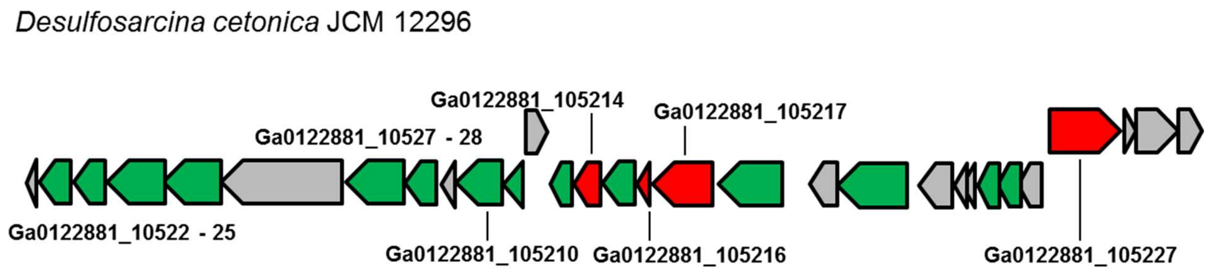

Fig. S2 Gene cluster showing specifically acetone-induced genes in *Desulfosarcina cetonica*. Most prominent genes are symbolized as red arrows and are annotated as acetolactate synthase (105227), a small and a large subunit of a methylmalonyl-CoA mutase (105216, 105217) and a 3-oxoacyl-[acyl-carrier protein] reductase (105214). Green genes are found to be expressed acetone-specifically (also in butanone- and isopropanol-grown, not in butyrate-grown CFE) by proteome analysis. These genes are annotated as an oxaloacetate decarboxylase, gamma chain (10522), a hypothetical protein (10523), an oxaloacetate decarboxylase beta subunit (10524), a propionyl-CoA carboxylase carboxyltransferase subunit (10525), a long-chain acyl-CoA synthetase (10527), acetyl-CoA C-acetyltransferase (10528), as well as a hydroxymethylglutaryl-CoA lyase (105210), a hypothetical protein (105211), a glyoxylase, beta-lactamase superfamily II (105213), LAO/AO transport system kinase (105215), an acyl-CoA hydrolase (105218), an anaerobic selenocysteine-containing dehydrogenase (105220), a two component transcriptional regulator, LuxR family (105224) and a histidine kinase-, DNA gyrase B-, and HSP90-like ATPase (105225).
